# Triggered: Qualitatively exploring structural and social drivers of firearm violence exposure among LGBTQ+ young adults of color in Detroit

**DOI:** 10.1016/j.socscimed.2025.118524

**Published:** 2025-08-27

**Authors:** Wesley M. Correll-King, Ini-Abasi Ubong, Dior Monro, Kalaan Scott, Sydney N. Strunk, J. Stephenson, Laura Jadwin-Cakmak, Avery Everhart, Kristi E. Gamarel

**Affiliations:** a University of Michigan Population Studies Center, 426 Thompson Avenue, Ann Arbor, MI, 48106, USA; b Department of Health Behavior and Health Equity, University of Michigan School of Public Health, 1415 Washington Heights, Ann Arbor, MI, 48109, USA; c Ruth Ellis Center, 5 Victor St, Highland Park, MI, 48203, USA; d Department of Geography, University of British Columbia, 1984 West Mall, Vancouver, BC, V6T 1Z2, Canada

**Keywords:** Firearms, Structural violence, Structural racism, Sexual minorities, Gender minorities

## Abstract

Firearm violence is a leading cause of injury and death among youth and young adults in the U.S. with notable inequities across race and ethnicity, geography, and gender. Lesbian, gay, bisexual, transgender, and queer (LGBTQ+) young adults are largely absent from firearms research. Guided by structural violence and Social Safety theory, we qualitatively explored structural and social influences on firearm violence exposure among LGBTQ+ young adults of color in Detroit, Michigan. Through analysis of in-depth interviews with 24 participants, we developed three themes aligned with this aim. First, participants’ accounts reflected how contemporary and historical structural racism in Detroit is the root cause of the firearm violence. Second, participants characterized firearms as a source of protection in the absence of structural safety. Finally, participants described how firearm violence against LGBTQ+ people is often an attempt to regain social status lost to structural violence. These themes indicate that structural racism in Detroit has unique impacts on LGBTQ+ young adults of color’s exposure to firearms and firearm violence. Future research with this community is needed to guide protective interventions and policy changes.

## 1. Introduction

Firearm violence is a critical public health issue affecting the safety and well-being of the general population (Grinshteyn and Hemenway, 2019; Semenza and Kravitz-Wirtz, 2024). Compared to peer countries, the U.S. has nearly 10 times the rate of firearm suicide and 25 times the rate of firearm homicide (Grinshteyn and Hemenway, 2019). Firearms play a dominant role in making injury the leading cause of mortality among youth and young adults (Bottiani et al., 2021; Roche et al., 2023; Wolf et al., 2024; Woolf et al., 2023). Race and ethnicity, gender, and geography are key sociodemographic predictors of firearm injury and mortality (Bottiani et al., 2021; Roche et al., 2023; Wolf et al., 2024). Black boys and young men living in economically depreciated urban areas are extreme outliers in risk for firearm homicide, and Indigenous and White boys and young men living in rural areas are at increased risk of firearm suicide (Bottiani et al., 2021; Roche et al., 2023).

Research has also examined community firearm violence exposure and its mental and behavioral health effects among youth and young adults (Bancalari et al., 2022; E. Lee et al., 2024). Community firearm violence encompasses community exposure (e.g., hearing gunshots), secondary exposure (e.g., a family member being injured with a firearm), and direct exposure (e.g., being threatened with a firearm) (Bancalari et al., 2022; Semenza and Kravitz-Wirtz, 2024). These experiences are associated with adverse mental and emotional health outcomes including loneliness, alienation, and post-traumatic stress symptoms, which can impact physical health by changing diet, substance use, and exercise behaviors (Semenza and Kravitz-Wirtz, 2024). As with firearm homicide and mortality, community firearm violence is most prevalent in economically depreciated urban areas, disproportionately affecting Black youth and young adults (Semenza and Kravitz-Wirtz, 2024).

Researchers have called for increased attention to systems, policies, and institutional practices that drive racial inequities in firearm homicide and community firearm violence (Buggs et al., 2023; Randolph et al., 2024). Studies in this vein have found that 1930’s redlining predicts contemporary firearm violence regardless of current neighborhood poverty levels (Benns et al., 2020; Dholakia et al., 2024). Furthermore, neighborhood socioeconomic disadvantage statistically explains more of the racial and ethnic inequities in community violence than household socioeconomic status among urban youth (Kravitz-Wirtz et al., 2022). These examples suggest that structural- and community-level exposures warrant increased attention in firearms prevention research.

### 1.1. *Firearm violence among LGBTQ*+ *youth and young adults*

While research on racial inequities in firearm exposure, injury, and mortality is expanding, firearms researchers have generally overlooked lesbian, gay, bisexual, transgender, and queer (LGBTQ+) communities (Conron et al., 2018; Correll-King et al., 2025). LGBTQ+ communities, particularly youth, experience inequities in intimate partner violence, suicide, and bias-related interpersonal violence, all of which may involve firearms (Bruner et al., 2019; Centers for Disease Control and Prevention, 2024; Di Giacomo et al., 2018; Kim and Schmuhl, 2021; Wirtz et al., 2020). Existing research indicates LGBTQ+ youth and young adults may be less likely to carry firearms and use them in suicides than non-LGBTQ+ youth and young adults (Harper et al., 2023; Ream, 2022). Additionally, LGBTQ+ adults are more likely to fear firearm violence victimization than non-LGBTQ+ adults, with LGBTQ+ people of color reporting greater levels of fear than White LGTBQ+ people (Conron and Tan, 2024). However, little is known about firearm violence impacting LGBTQ+ people of color (Correll-King et al., 2025). This study seeks to address this gap in knowledge by examining the structural- and community-level determinants of firearm violence exposure among LGBTQ+ young adults of color.

### 1.2. Theoretical frameworks

Two frameworks guide this research: structural violence and Social Safety Theory. Structural violence refers to the use of power to construct systems, policies, and practices that advantage some groups by depriving others of basic needs (Galtung, 1969). In response to increasing inequities in firearm injury, firearm violence researchers have explicitly called for greater attention to how structural violence and community firearm violence mutually influence each other (Buggs et al., 2023). This requires contextualizing inequities within the historical legacy of structural racism, including the social control projects of colonization, slavery, segregation, and mass incarceration that constrain and exploit communities of color to promote economic and cultural security for White people (Boeck et al., 2020; Metzl et al., 2021; Randolph et al., 2024; Uzzi et al., 2024). Adopting a structural violence framework avoids replicating narratives of individual or cultural criminality and blameworthiness for firearm violence used to support “public safety” prevention approaches that strengthen the criminal-legal system and uphold the conditions in which racial inequities in firearm violence occur (Buggs et al., 2023). Therefore, in this study, we explicitly seek to identify and interrogate how structural violence manufactures the conditions precipitating firearm violence impacting LGBTQ+ young adults of color.

Social Safety Theory complements and extends structural violence by considering how social threat *or* the absence of social safety activates neural pathways and inflammatory responses that drive human behavior and impact health (Slavich et al., 2023). Social safety refers to authentic and dependable social inclusion, belonging, and connection whereas social threat refers to conflict, isolation, devaluation, rejection, and exclusion (Slavich et al., 2023). Social Safety Theory may be especially relevant to understanding LGBTQ+ health inequities as anti-LGBTQ+ stigmas (i.e., heterosexism, cissexism) reduce exposure to social safety and increase exposure to social threat (Diamond and Alley, 2022). The absence of social safety has been strongly implicated in suicide deaths, attempts, and ideation among LGBTQ+ people (Diamond and Alley, 2022), which are of particular relevance to our study on firearms.

Social Safety Theory may also be used to explain individual firearm use and exposure to firearm violence. Research with White men indicates that firearm ownership may be a self-protective coping mechanism used to manage perceived threats to their status within racial, gender, and socioeconomic hierarchies by promoting a sense of safety, power, and control (Buttrick, 2020; Higginbotham, 2024; Scaptura and Boyle, 2022). Recent research seeking to understand firearm use among Black Americans has examined how structural racism drives use of firearms for self-protection and for building and maintaining power in economically depreciated urban communities of color, driving racial inequities in firearm injury and mortality (Higginbotham, 2024). This research contextualizes firearm violence within the absence of effective social programs and maintenance of racially stratified systems that structure economic opportunity—or, in Social Safety Theory terms, in the omnipresence of social threat and absence of social safety (Randolph et al., 2024).

In this way, both LGBTQ+ people of color’s firearm use and anti-LGBTQ+ firearm violence can be viewed as behavioral responses to absence of social safety or presence of social threat. Black firearm ownership has been described as a means of collective empowerment, safety, and resistance to White supremacy and thus an adaptive response to centuries of structural violence levied at Black communities (Higginbotham, 2024). The strengthening backlash against LGBTQ+ human rights and increasing political, legal, and physical attacks on LGBTQ+ people may similarly shape LGBTQ+ people of color’s views on or use of firearms (Flores et al., 2021; Kehoe, 2020). Simultaneously, like White men’s use of firearms to maintain their position atop social hierarchies, bias-motivated anti-LGBTQ+ violence may stem from perpetrator’s desires to alleviate the perceived threat of LGBTQ+ people’s disruption of cisheteronormativity (Morgenroth and Ryan, 2021).

### 1.3. Setting

This study is set in Detroit, Michigan. Detroit is a highly segregated and majority Black rustbelt city whose demographic divisions have been shaped by redlining, predatory mortgage lending, and government corruption (Newman et al., 2019). Contemporary Detroit exemplifies how neoliberal urbanism and “eco-conscious” redevelopment collide to reinforce racial inequities via gentrification, corporatism, and racial capitalism (Clement and Kanai, 2015; Montgomery, 2015; Safransky, 2014; Smith, 2017).

In 2013, the city implemented the Detroit Future City Plan to address decades of population decline, financial mismanagement, and economic deterioration (Clement and Kanai, 2015; Griffin et al., 2014; Markus and Krings, 2020). Its adoption had strong implications for how the city assesses and pursues firearm violence reduction. Efforts have included strengthened police surveillance via a citywide network of CCTV cameras, improved ballistic evidence technologies, and targeted deterrence programs (Circo, 2020; Circo et al., 2021; De Biasi, 2024). Other interventions have focused on remediating vacant buildings and developing greenspace (Breetzke et al., 2020; Jay et al., 2019; Larson et al., 2019; Regan and Myers, 2020).

From 2011 to 2019, overall firearm violence victimization in Detroit decreased (Circo et al., 2021). However, pediatric firearm injuries have been rising since 2013, and youth living in Detroit are more likely to hear gunshots and witness shootings than peers in neighboring non-urban areas (Borg et al., 2020, 2023). Moreover, overall firearm violence in Detroit significantly increased during the COVID-19 pandemic (De Biasi et al., 2023). In 2022, Detroit had the second highest rate of firearm deaths among major U.S. cities (51 per 100,000 people) (Urban Health Collaborative, 2022).

As in other cities, bias-motivated homicides of trans women of color in Detroit have been increasingly documented over the last several years (Lantz et al., 2024). A list of convictions achieved by a Detroit-based legal advocacy group suggests that firearms are frequently used in homicides, assaults, and robberies against cisgender gay men and trans women (Fair Michigan, 2022). However, to our knowledge, no efforts have been made to document firearm violence impacting LGBTQ+ people in Detroit beyond reporting of isolated incidents.

### 1.4. Study purpose

This study examines the structural and social determinants of LGBTQ+ young adults of color’s experiences with firearms and firearm violence in Detroit. We intend for these findings to draw public health’s attention to firearm violence impacting LGBTQ+ people of color and guide future firearm injury prevention initiatives and research.

## 2. Methods

This qualitative study is part of a larger formative research effort to understand and address firearm violence among LGBTQ+ young adults of color in Detroit. We partnered with two community-based organizations serving trans people of color to design and conduct this research. The University of Michigan Institutional Review Board approved all study procedures.

Individuals were eligible for this study component if they met the following criteria: (1) identified as LGBTQ+; (2) identified as any race or ethnicity except non-Latine White; (3) were between 15 and 29 years old; (4) had been victimized by or witnessed firearm violence, including secondary and community violence; (5) resided in or near Detroit; (6) could provide informed assent/consent; and (7) were English-speaking. Our partnering organizations recruited participants via flyers, social media, and word of mouth. Interested individuals were asked to contact a member of the study team who then conducted an eligibility screener via phone, Zoom, or in person.

Semi-structured interviews occurred from February–August 2023 in private rooms at the Ruth Ellis Center, a health and social services center serving LGBTQ+ youth. We collaboratively developed the interview guide with our partnering organizations and experts in firearms research (see Supplementary Material). Topics included community perceptions (e.g., “What do you think causes gun violence within your community?“), personal experiences (e.g., “Can you tell me about the first time you saw a gun in real life?“), and areas for future firearm injury prevention research and program development (e.g., “What do you think organizations could do to better help LGBTQ+ youth and young adults?“). Interviews were audio-recorded on external devices. Recordings were transcribed, de-identified, and quality checked. Participants received a $40 incentive, meal, and list of LGBTQ+ -affirming mental health and social services with the option for facilitated referrals. All participants received a wellness check via phone 48 h after their interview.

Three trans researchers trained in qualitative interviewing conducted the interviews. Because of the difficult subject material, interviewers regularly debriefed with other members of the study team using team-created materials for processing trauma activated during the research process. Interviews paused during April and most of August because of the psychological toll the process took on interviewers.

The transcripts were iteratively analyzed using methods grounded in reflexive thematic analysis (Braun and Clarke, 2022). First, two team members (KEG, LJC) began preliminary analysis by identifying influences on firearm violence exposure across all levels of the social-ecological model. Through discussion with other members of the team including interviewers (IAU, DM, KS, JS), they used these notes to generate and apply inductive codes to the full dataset.

For this analysis, we focused on structural and community-level influences on firearm violence. After reviewing the existing codes and notes, the lead analyst for this portion of the project (WCK) repeatedly read each transcript and took detailed notes capturing apparent and latent meaning in the data related to this research aim. He then drafted several candidate themes organized around central concepts that reflected shared meaning in the data. Through discussion with other members of the study team (KEG, IAU), he revised and organized the candidate themes into a concise, coherent draft set of themes and emblematic quotes. Two team members (IAU, WCK) presented these draft themes to the full study team, which included members of partnering organizations and all of the interviewers. All provided feedback on the importance, relevance, and credibility of each theme and supporting quotes, including the extent to which they believed the analysts’ interpretation of the data was valid. The results were then revised and finalized, prioritizing feedback from study team members closer in social position to the study participants (e.g., from Detroit, Black, LGBTQ+). We did not quantify how many participants endorsed each theme as reflexive thematic analysis prioritizes depth and relevance over frequency (Braun and Clarke, 2021).

## 3. Results

Participants (N = 24) ranged from 18 to 29 years old and most (n = 20) were non-Latine Black (Table 1). Most participants were trans feminine or trans women of various sexual orientations (n = 10) or cisgender gay, bisexual, or pansexual men (n = 9). Below, we present three themes we developed ordered from broad to specific. The first discusses structural racism as a root cause of firearm violence in Detroit and is heavily based in participants’ early life experiences. The second describes structural and community-level influences on participants’ perceived risk of violence and the role firearms play in managing that risk. Finally, the third highlights how structural violence drives firearm violence against LGBTQ+ people. Aligned with reflexive thematic analysis, we provide illustrative quotes to support each theme and interpret the quotes within their context (e.g., then-current community events, previous parts of the interview) to clarify their meaning and connection to the theme (Braun and Clarke, 2022).

### Theme 1. Structural racism in Detroit is the root cause of the firearm violence participants experience.

Most participants grew up in Detroit, and many described how connections between persistent community poverty and firearm violence manifested over the course of their lives. Quintell immediately identified poverty as a dominant contributor, saying, “*What causes it? I’m gonna start at poverty. Have to, unfortunately. Like I got friends that’s not bad people, [but they’re] criminals. And people do things for reasons, and maybe they either feed their baby, pay their bills. That’s often what it is in Detroit, poverty.”* Quintell’s distinction between “criminals” and “bad people” reflects his understanding of how poverty creates circumstances in which illegal and even violent activity facilitates survival.

Participants’ narratives highlight how structural racism fosters cycles of violence within predominantly Black communities by manufacturing conditions of economic deprivation. This includes diminished access to high quality education and employment opportunities and the resultant proliferation of underground economies (e.g., sex work, drug trade). Comparing community firearm violence to the results of a science experiment, Liana said, “*If you put a bunch of mice in one specific area, but you don’t give them enough food, you don’t give them enough resources, of course they’re gonna attack each other … This goes back to Jim Crow laws and Reconstruction and all that when they was building all these ‘Ok, this is where 8 Mile is gonna cross that [road] … This is the White side, this is the Black side.’ I feel like all that plays into the gun violence [and] just crime rate alone because we don’t have shit. And so we turn on ourselves. We turn on each other.”*

Liana’s analogy directs attention to how policymakers and urban planners in Detroit have divested from Black communities to create and maintain resource scarcity, fueling community violence. Other participants used similar language to describe how structurally racist socioeconomic, educational, and employment opportunities contribute to firearm violence saying, “*it’s almost a setup to our community as Black people … you’re set up for failure in the hood*” (Simon) and “*people are products of their environments … [firearm violence] really don’t be an act of hate, it really be an act of hunger*” (Farid).

Some participants offered examples of how poverty contributed to traumatic experiences and early childhood exposure to firearm violence. For example, Jolie shared, “*The situation* [my ex-boyfriend’s] *family was in, it was either work as a young kid doing what they needed to do, or y’all froze in the house with no heat … No child should ever have to go through something like that ‘Oh, you’re 12 years old? It’s time for you to go out there and start shooting niggas, selling weed, and running from the police.’”* Other participants shared how their parents facilitated their first exposure to firearms. Malcolm reflected on how he learned that “*the ultimate display of power and protection is a gun*” when his father taught him how to shoot following a home robbery.

Overall, many participants felt that a desire to preserve social standing and safety was behind the cyclical, intergenerational patterns of firearm violence in their communities, not just that perpetrated by or against LGBTQ+ people. Participants suggested that firearm ownership confers a sense of power, pride, and superiority over others. For instance, Vaughn dismissed efforts to end firearm violence saying, “*Nobody’s putting the guns down. Everybody wants somebody else to feel inferior, they wanna feel like they’re [on top].”* Placed in the context of historical structural racism, firearm ownership among people of color in Detroit can be understood as a way to build social status within a persistently anti-Black environment.

### Theme 2. Firearms as a source of protection in the absence of structural safety.

Nearly all participants expressed negativity towards firearms and supported policies intended to restrict their access and improve firearm safety. Even those most opposed to firearms characterized them as necessary sources of protection for many LGBTQ + people of color. Tasha repeatedly described her firearm as “*a killing machine*” and expressed serious internal conflict over owning it. Ultimately, she felt that “*in today’s time, people [in my community] definitely need them”* and explained how firearms can offer protection against anti-LGBTQ+ violence or violence from sex work clients and intimate partners.

Participants described how schools, workplaces, media, and government policies limit LGBTQ+ people’s access to resources and broadly signal that they are not worthy of equal protection. For example, Malcolm connected violence many trans women experience during sex work to the scarcity of other means of economic survival: “*A lot of girls have experience of being raped by their date or things of that nature because he has a gun … And a lot of girls do [sex work] because this is a survival thing for them. ‘Cause they don’t have the protection or we don’t always have the protection to go out and get a 9*–*5 and have respect in the workplace.”* A former sex worker himself, Malcolm’s comments suggest that lack of social safety and blatant employment discrimination contributes to trans women’s engagement in survival sex work, driving exposure to firearm violence.

Participants identified trans women and, to a lesser extent, feminine queer men within their communities as most at risk for experiencing violence and felt that this increased risk justified firearm ownership. For example, Cairo shared that LGBTQ+ people might want to own firearms because they, “*just want to remain safe because of the stuff that goes on in our city or just in the world, period. We watch the news all the time and see different people getting harmed, different people getting shot … A person may see you out, and they automatically looking at you as kind of softer than who they are because you’re gay.”* The sense of threat Cairo feels is both particular to Detroit (“our city”) and a more universal experience for LGBTQ+ people of color. His comments are likely in relation to the then-recent 2023 murder of O’Shae Sibley, a Black teen who was stabbed to death in Brooklyn, NY in a homophobic hate crime. Cairo’s remarks reflect how the effects of anti-LGBTQ+ violence can reverberate across place to effect perceptions of social threat and safety and influence firearm use.

Gabrielle commented on trans women’s firearm use in relation to narratives suggesting the world has improved for trans people. She said, “*I feel like every trans girl should have a gun, because for one, our safety. People already don’t like us. I feel like the world really hates us. I don’t care how we are ‘changing the world’ … The world actually really hates us. Especially the men that claim they really fuck with us … We need guns everywhere we go.”* For Gabrielle, superficial progress towards trans inclusion does not change how cissexism and transmisogyny continue to threaten trans women’s physical safety. The absence of structural and social safety makes firearms the most effective means of ensuring personal safety against the multitude of threats she experiences both in intimate settings and in the world at large.

### Theme 3. Firearm violence against LGBTQ+ people is often an attempt to regain social status lost to structural violence.

When interviewers asked participants to identify causes of firearm violence committed against LGBTQ+ people, most participants described instances that blur the line between intracommunity (i.e., between LGBTQ+ community members) and intercommunity (i.e., between non-LGBTQ+ and LGBTQ+ individuals) violence. Specifically, participants discussed how trans women and visibly queer men are at heightened risk of firearm violence perpetrated by cisgender men who are attracted to and/or have had sexual relationships with such people but who do not consider themselves part of LGBTQ+ communities. Farid shared, “*You have to take into consideration that these bisexual young men are having these feelings that they don’t know how to control, and they are killing people in the community.”* While Farid labeled these perpetrators as bisexual, he also distinguished them from “the community.” For Farid and others, cisgender men’s sexual activities with or attraction to people who are not cisgender women do not necessarily make them part of the LGBTQ+ community.

Individuals with these types of complex relationships to LGBTQ+ communities were the most common perpetrators of anti-LGBTQ+ firearm violence that participants or their peers experienced. Participants described this violence as rooted in perpetrators’ self-protective desires to preserve their sense of social safety. For example, Cairo shared, “*But the trade is living a secret life. So, they want their secret to be their secret. And at the risk of you putting their secret out, they might shoot you.”* Farid and Cairo’s comments suggest that these perpetrators (“the trade”) are attempting to preserve their standing in heterosexual communities by concealing a stigmatized attribute (i.e., their attraction to men or trans women), giving them a sense of power, control, and safety.

Malcolm offered a similar perspective when explaining why firearm homicides against trans people are so common. He said, “[It’s] *something that the other person was struggling with … A lot of times they lose it, and they use* [trans people] *as a scapegoat because they know we don’t get the justice that we’re supposed to get.”* His comment illuminates how denigration of trans people in the criminal-legal system allows perpetrators of anti-trans firearm homicides to justify their actions. Cisgender men who are attracted to trans people can use firearm violence to manage perceived or anticipated threats to their social safety because trans people—particularly trans women of color— are so devalued as to be acceptable targets of violence.

Several trans participants discussed how cisgender men of color are the typical perpetrators of violence against trans women of color in their communities. Hadlee shared about a recent firearm homicide of a trans woman of color committed by a Black cisgender man at an LGBTQ+ club, saying, “*Most of the time it be our own people … It’s like, come on, already people out here struggling and homeless and things like that to the point of they don’t have nothing. And it be like some people don’t have nothing to live for and take care of.”* Hadlee’s use of “our own people” suggests she views the perpetrator as a member of her community. Attributing his actions to “nothing to live for and take care of” underscores how lack of socioeconomic opportunity, purpose, and social connection can fuel community firearm violence.

Similarly, Vaughn implicated lack of sustainable employment opportunities when discussing Black cisgender men’s perpetration of firearm violence. Referring to racist hiring discrimination, he said “*Some people can’t even get jobs off their names,”* and later in his interview elaborated on the gendered nature of racial employment gaps by saying, “*You see more Black women than you see Black men* [with jobs] … *They always try to put all the Black men in the factories if anything and overwork their ass. They get home and be fucking tired and angry*.” Vaughn’s comments contextualize incidents like the one Hadlee described within gendered experiences of economic marginalization that systematically deny social status, power, and safety to Black men.

While most of the instances of firearm violence participants described were characterized by blurred community lines between perpetrator and victim(s), some instances were unquestionably intracommunity. Participants also attributed intracommunity firearm violence to gendered experiences of economic marginalization. Firearm violence or threats thereof occasionally occurs in LGBTQ+ community spaces, most often resulting from arguments over romantic partners, drugs, or sex work. For instance, Jolie shared, “*When I used to walk the stroll at Palmer Park, prostitution,* [firearm violence] *could be over territory, space. Like I done had to fight a couple people. Me being new, I didn’t know whose spot was whose spot.”* While no participants discussed specific instances of firearm violence between sex workers, many shared that sex workers frequently carried firearms in part to secure access to clients. In this way, firearms help confer access to financial resources in a context in which LGBTQ + people of color are denied other economic opportunities.

## 4. Discussion

The themes developed from this analysis illustrate how structural racism in Detroit shapes the physical, social, economic, and political environment, influencing how the LGBTQ+ young adults of color we interviewed experience firearm violence. Findings revealed how a profound absence of social safety strengthens the perceived importance of owning firearms for individual and community protection. Our analysis identified perpetrators’ latent needs to preserve their social standing, economic resources, safety, and sense of power as important proximal causes of firearm violence. Ultimately, our findings begin to uncover how structural violence creates conditions in which these social and psychological resources are threatened and in which LGBTQ+ lives are deemed expendable collateral for managing those threats.

Our findings corroborate literature suggesting that firearm violence in impoverished urban communities of color results from structural violence rooted in structural racism (Boeck et al., 2020; Uzzi et al., 2024). Integrating a structural violence perspective contextualizes participants’ exposure to firearm violence by directing attention to the systems, policies, and practices that prevent marginalized groups from meeting their basic needs while maintaining power and access to resources for dominant groups. Participants directly referenced historical racist practices (e.g., redlining), limited educational and socioeconomic opportunities, and community-level poverty in Detroit when discussing the main causes of community firearm violence, echoing results from other similar studies (Boeck et al., 2020; Gobaud et al., 2024; Uzzi et al., 2024).

These factors mirror structural and community level determinants of firearm violence victimization identified in studies of other Great Migration cities, most of which center cisgender Black men’s experiences (Burrell et al., 2021; Dillard et al., 2023; Walsh et al., 2024). During the Great Migration, millions of Black people living in the south moved to industrial centers in the north and west in search of greater economic, social, and political freedom (Berlin, 2010). Like in Detroit, structurally racist practices in these cities including police hyper-surveillance of communities of color (Bernstein, 2022), racist discrimination (Burrell et al., 2021), and inadequate government funding for community-based social services and violence prevention programs (Carswell, 2022; Fleming et al., 2021) have maintained high levels of racial segregation and concentrated poverty. In these environments, firearms are figurative and literal means of ensuring personal and collective safety (Higginbotham, 2024; Oliphant et al., 2019), economic survival (Beck et al., 2019), and self-respect (Outland, 2019; Warner et al., 2022). As in other studies with urban young adults of color, participants in our study described learning in childhood that firearms can offer protection from violence, confer social status, and be used to access economic resources (Fontaine et al., 2018). Social Safety Theory suggests that beginning in early life, individuals form cognitive representations of the world that determine to what extent they are primed to perceive safety and threat in novel social situations (Slavich et al., 2023). Previous research indicates that exposure to firearm violence in childhood can lead to difficulty envisioning positive future outcomes and greater fear responses, which in turn are associated with carrying firearms in adolescence (Bancalari et al., 2022; D. B. Lee et al., 2020). Beliefs that firearms are necessary for protection suggests that the lessons participants learned in childhood were reinforced over time, strengthening the role of firearms in their social safety schemas.

While some of our themes are consistent with studies of other marginalized urban populations, our analysis builds on this literature by illuminating how intersectional structural racism, cissexism, and heterosexism shape LGBTQ+ people of color’s access to social safety and vulnerability to firearm violence (Bowleg et al., 2023; Crenshaw, 1991; Wesp et al., 2019). In particular, participants described how schools and workplaces in Detroit can be socially threatening for LGBTQ+ people of color. Previous literature has discussed how racist and cissexist practices in schools manufacture social threats such as bullying and disproportionate discipline for LGBTQ+ youth of color, driving school dropout (Aguilar et al., 2024; Rosentel et al., 2020). Similar dynamics manifest in LGBTQ+ people of color’s experiences in the workplace (Sears et al., 2024). Thus, these findings suggest that the absence of social safety in these institutions has lasting consequences for LGBTQ+ people of color’s socioeconomic position and firearm violence exposure.

Furthermore, our results suggest that intersectional oppression shapes the experiences, beliefs, and behaviors of perpetrators of firearm violence against members of this community. Cisgender men perpetrated nearly all firearm violence participants described. Sociologists have argued that limits to economic opportunity for men blocks avenues for embodying the “masculine provider” role, threatening self-perceptions of masculinity (Carlson, 2015; Cassino and Besen-Cassino, 2020). The “masculine protector” role then increases in cultural and personal significance, shaping positive attitudes towards firearms and increased firearm use (Warner et al., 2022). This phenomenon is specifically noted among men who perceive their individual socioeconomic struggles as threatening to their larger cultural or racial group (Borgogna et al., 2022; Higginbotham, 2024; Scaptura and Boyle, 2022). Black cisgender men in Detroit may be part of this phenomenon as racial capitalism constrains their access to economic opportunity.

Participants’ accounts add critical nuance to violence prevention research focused on understanding influences on men’s firearm violence perpetration, including gender-based violence. The perpetrators of most firearm violence our participants described were using firearms for protection against the perceived threat of LGBTQ+ people’s subversion of cissexist and heterosexist norms to their status within hegemonic masculinity. This subversion destabilizes cultural and individual senses of masculinity already imperiled by the constrained economic opportunities for cisgender men of color in Detroit. As a result, cisgender men— particularly those confronting internalized anti-LGBTQ+ bias— may display aggression, hatred, and violence in attempt to reduce this perceived threat rather than reimagine or redefine masculinity (Diefendorf and Bridges, 2020; Garrett-Walker et al., 2019). Incorporating content designed to reduce anti-LGBTQ+ bias into existing gender-based violence prevention programs which focus on developing positive masculinity may help address firearm violence perpetration (Pérez-Martínez et al., 2023).

This is among the first firearms studies to center LGBTQ+ young adults of color (Correll-King et al., 2025). Previous work has examined impacts of the Pulse nightclub shooting on LGBTQ+ people of color (Kline, 2022; Molina et al., 2019; Ramirez et al., 2018). While some participants in our study alluded to the wide-ranging, indirect impacts of publicized anti-LGBTQ+ violence like the Pulse shooting, most focused on incidents of firearm violence they were exposed to directly, secondarily, or through their social networks. These experiences have not been previously represented in public health literature and suggest that anti-LGBTQ+ stigmas make sexual orientation and gender identity salient and understudied predictors of firearm injury and mortality (Correll-King et al., 2025).

In calls for greater LGBTQ+ inclusivity in firearms research, nationally prominent LGBTQ+ research and advocacy organizations’ central focus has been on suicide prevention (The Trevor Project, 2024). However, participants in our study rarely discussed suicide. As with cisgender, heterosexual populations, this may be because firearms are a less common means of suicide among Black and Latine LGBTQ+ people as they are among other groups (Boeck et al., 2020). As research on LGBTQ+ communities and firearms expands, focusing primarily on suicide prevention may neglect Black and Latine LGBTQ+ communities’ needs.

This study is intended to lay groundwork for future research informing community-level interventions and policy changes to prevent firearm injury among LGBTQ+ young adults of color. Most participants did not articulate solutions to firearm violence when asked, and we were therefore unable to develop solution-oriented themes. Nevertheless, our findings suggest that interventions focused on strengthening socioeconomic opportunities, promoting LGBTQ+ social safety, reducing anti-LGBTQ+ stigma, and addressing structural racism may be promising starting places for preventing firearm violence against LGBTQ+ young adults of color (Abreu et al., 2022; Bailey et al., 2017; Gould et al., 2025; Restar et al., 2025; Rowhani-Rahbar et al., 2022).

### 4.1. Limitations

This study has several important limitations. First, because participants were recruited through a trusted community-based organization, our sample may over-represent young adults who are more socially connected or open about their identities. Individuals who are not out, who socialize primarily in heterosexual spaces, or who are disconnected from LGBTQ+ networks may have different patterns of firearm violence exposure, including less bias-motivated firearm violence. Second, while our sample reflects our partnering organization’s demographics, findings do not capture the firearm experiences of other LGBTQ+ young adults of color including cisgender women or those from different racial or ethnic communities. Third, because our sample did not include minors, our findings may not reflect how firearm violence is experienced by LGBTQ+ adolescent, which may be more strongly shaped by familial or school environments. Finally, as with all qualitative research, findings should be interpreted as illustrative rather than generalizable. Thematic analysis is an interpretive process shaped by the positionality of the research team. While our team included members with social positions similar to those of participants, other researchers might arrive at different insights from the same transcripts.

## 5. Conclusion

By centering the experiences of LGBTQ+ young adults in Detroit, we reveal how structural racism profoundly influences individuals’ encounters with firearms and firearm violence and fosters perceptions that firearms are necessary for protection. Intersecting structural racism, cissexism, and heterosexism exacerbate economic vulnerabilities and limit access to social safety. Given the lack of violence prevention interventions that intentionally include LGBTQ+ young adults of color, we call for multisector collaborations between firearms injury prevention researchers, LGBTQ+ health researchers, community leaders, and young people focused specifically on enhancing social safety and reducing firearm injury.

## Supplementary Material

1

## Figures and Tables

**Table 1:** Characteristics of participants, N = 24.

Pseudonym	Age	Race and Ethnicity^a^	Gender	Sexual Orientation
Aria	22	Latine White	Trans feminine	Bisexual
Bryce	26	Latine Black	Man (cisgender)	Pansexual
Cairo	18	Black	Man (cisgender)	Bisexual
David	26	Black	Trans masculine	Straight
Essence	26	Black	Trans feminine	Gay
Farid	26	Black	Man (cisgender)	Gay
Gabrielle	21	Black	Trans feminine	Pansexual
Hadlee	29	Black	Trans feminine	Gay
Imani	26	Black	Trans feminine	Pansexual
Jolie	20	Black	Trans feminine	Gay
Kyra	25	Black	Woman (transgender)	Bisexual
Liana	29	Black	Woman (transgender)	Straight
Malcolm	29	Black	Man (cisgender)	Pansexual
Nuri	24	Black	Two-Spirit, Nonbinary	Bisexual, Straight
Omarion	26	Black	Man (cisgender)	Gay
Parker	25	Black	Man (cisgender)	Bisexual
Quintell	26	Black	Man (transgender)	Not reported
Rome	27	Black	Not reported	Bisexual
Simon	25	Black	Trans masculine	Straight
Tasha	28	Black	Trans feminine	Gay
Vaughn	29	Black	Man (cisgender)	Pansexual
Xavier	23	Not reported	Man (cisgender)	Bisexual
Yara	25	Not reported	Woman (transgender)	Straight
Zion	26	Black	Man (cisgender)	Gay

a Non-Latine unless noted.

## Data Availability

The data that has been used is confidential.

## Appendix A. Supplementary data

Supplementary data to this article can be found online at https://doi.org/10.1016/j.socscimed.2025.118524.
