# Supplementary material for: Triggered: Qualitatively exploring structural and social drivers of firearm violence exposure among LGBTQ+ young adults of color in Detroit
Source: Soc Sci Med. Author manuscript; Available in PMC 2026 Jul 6. (PMC13335610; doi:10.1016/j.socscimed.2025.118524)
Supplement: 1 [file NIHMS2182143-supplement-1.docx]

SUPPLEMENTARY MATERIAL

Abbreviated Interview Guide

**Ii. Warm-Up/Building Rapport**

1. **So, to get started, tell me a little bit about yourself.**

- How do you usually spend your time?
- What are you passionate about?
- What do you do for fun?

1. **How long have you lived in the Detroit Metro Area?**

- *[If living in city a long time]* How do you like living here?
- *[If living in city less time]* What brought you here?

1. **Tell me a little about how you identify within the LGBTQ+ community.**

- *What pronouns do you use?*

**III. COMMUNITY PERCEPTIONS**

**Thanks for telling me a little about yourself. So, now I want to learn more about your thoughts on guns in the LGBTQ+ community.**

1. **I keep saying “LGBTQ+ community.” Can you tell me a little about what you consider to be your LGBTQ+ community in Detroit?**
   - *If they talk about specific people, like friends or partners*
     1. How did you meet?
     2. Where do you usually spend time with them? In ballroom, community activism, or other places?
   - *If they talk more generally about places, events, or groups*
     1. What made you start going there?
     2. What have you enjoyed about them?
   - *Probe about race, gender, sexuality since LGBTQ+ is broad*
     1. How, if at all, does race or ethnicity play into who you consider your community?
     2. Additional probes could include: gender, sexuality, geographic location, socioeconomic class, religion/faith, immigration status, relationship status
2. **People have a lot of different opinions about owning guns or carrying guns. How do you feel about owning guns or carrying them?**
3. **How do you think people in your local LGBTQ+ community feel?**
   - Why do you think some LGBTQ+ people might own guns?
     1. How, if at all, are these reasons different from the reasons why straight, cisgender people might own guns?
   - Why do you think some LGBTQ+ people might NOT own guns?
     1. How, if at all, are these reasons different from the reasons why straight, cisgender people might NOT own guns?
   - How do you think people in your local LGBTQ+ community feel about gun safety?
     1. Gun safety can refer to owning guns, gun storage, licensing etc.

1. **Gun violence is a serious issue for many people in Detroit. What have you heard LGBTQ+ people you know say about it?** [Getting shot, worrying about getting shot, being threatened with guns?]
   - Gun violence can also come from intimate partners, hookups, or dates. What have you heard LGBTQ+ people of color say about guns and intimate partner violence?
   - Gun violence also includes suicide and suicide attempts where people use guns. What have you heard LGBTQ+ people of color say about guns and suicide?
   - Gun violence can come from the police. What have you heard about LGBTQ+ people of color say about gun violence or intimidation with guns by the police?
   - Gun violence can come from white supremacists, people who hate LGBTQ+ people, and others. What have you heard LGBTQ+ people of color say about this?
2. **What do you think causes gun violence in your community?**

- Within your community?
- Against your community?
- Specifically, against Black trans people?
- How, if at all, are the causes different for transgender and non-binary people versus cisgender people?
- How, if at all, are the causes different for masculine presenting versus feminine presenting people?

**IV. PERSONAL EXPERIENCES**

**Thank you for sharing. Now I’d like to ask you a bit about your own experiences with guns and gun violence. This does not have to be limited to your experiences within the LGBTQ+ community. This topic can be very difficult for people to talk about, so please let me know if you would prefer to skip a question. I will not ask about any details like people’s names or exact locations. You can be as general or as specific as you would like.**

1. **Growing up, what were some of the messages you got from your family about guns?**
   1. How did these messages change as you got older?
   2. How did these messages compare to messages you got from peers? From the media?
2. **Everyone taking part in this study has been exposed to gun violence. Thinking back, can you tell me about the first time you saw a gun in real life?**
   1. How old were you? Who was with you?
   2. What was going on?
   3. What do you think this taught you about guns?
3. **I’d like to hear about [an/another] incident you witnessed or experienced involving gun violence that sticks out in your mind. This can be related to your LGBTQ+ identity, police violence, or any situation you think is relevant. Without telling me anything you’re not comfortable sharing, can you give me a summary of what happened?**
   1. How did you feel? Do you remember what you were thinking? How, if at all, did this incident impact your life afterwards?
   2. How do you think the situation may have been resolved if there were no guns involved?
   3. What made you choose to tell me about this incident over others you may have experienced?
   4. Is there anything else you’d like to share about this incident that may help us understand gun violence against LGBTQ+ youth and young adults? What about specifically for LGBTQ+ youth and young adults of color?
   5. Is there another incident you would be comfortable sharing about?
4. **When you know someone around you has a gun, how does that make you feel?**
   1. How is it different when you are…
      1. With people who share your identities?
         1. How is that different when you are with people who don’t share your identities? Or with people who share some of your identities?
      2. In a public space like a bar or a ball versus someone’s home?
      3. With people who are drinking, using marijuana, or using other drugs?
   2. How does it change how you act?
   3. How is it different depending on who has the gun? (Close friend or family vs. someone you don’t know vs. police, etc.)

**V. POTENTIAL STEPS FORWARD**

**Now I would like to talk with you about how to reduce gun violence within and against Detroit’s LGBTQ+ community.**

1. **Earlier, you talked about how you think gun violence might be caused by…[insert cause here from Section 3, Question 4 here]. What would need to change for that to no longer be an issue?**
   1. *Repeat for remaining causes identified in Section 3, Question 4*
2. **If you were the mayor of Detroit, what would you do to make Detroit safer for LGBTQ+ people of color?**
3. **There are organizations in Detroit working to address gun violence. What do you think these organizations could do to better help LGBTQ+ youth and young adults? What specific services could be provided for LGBTQ+ youth and young adults?**
4. **These interviews are the first step of a research project to understand experiences and needs. When I talk about research, in these interviews we’re trying to better understand your experiences with gun violence beyond the numbers and statistics. Research includes our process of translating your experiences and tracking common themes.**
   1. What topics did we not cover today?
   2. What should we explore further?
   3. What else should we be asking about?
   4. Are there experiences we’re missing out on that should be included?
5. **What types of research, if any, would you like to see that focus on gun violence among LGBTQ+ youth and young adults of color?**
   1. What should be the topics or focus?
   2. What would you not want to see?
   3. The LGBTQ+ community is quite broad. Would you prefer to have the focus broad or narrow? Why or why not? How?

**VI. conclude the interview**

**Thank you so much for taking the time to talk to me today.**

1. Is there anything else that you would like to share with me?
